# Data for Genetic Analysis Workshop (GAW) 15, Problem 1: genetics of gene expression variation in humans

**DOI:** 10.1186/1753-6561-1-s1-s2

**Published:** 2007-12-18

**Authors:** Vivian G Cheung, Richard S Spielman

**Affiliations:** 1 The Children's Hospital of Philadelphia, 3615 Civic Center Boulevard, Philadelphia, Pennsylvania 19104, USA; 2 Department of Genetics, University of Pennsylvania School of Medicine, 415 Curie Blvd, Philadelphia, Pennsylvania 19104-6145, USA; 3 Department of Pediatrics, University of Pennsylvania School of Medicine, 3516 Civic Center Blvd, ARC 516, Philadelphia, Pennsylvania 19104, USA

## Abstract

Here we describe the data provided for Problem 1 of Genetic Analysis Workshop 15. The data provided for Problem 1 were unusual in two ways. First, the phenotype was the level of gene expression for each gene, not a conventional phenotype like height or disease, and second, there were more than 3500 such phenotypes. Natural variation in gene expression was a new idea in 2004 when these data were collected and published. Because the phenotypes were measured in members of 14 Centre d'Etude du Polymorphisme Humain (CEPH) families, there was an opportunity for linkage mapping on a very large scale. For this purpose, 2882 single-nucleotide polymorphism genotypes were also provided for each family member.

## Background

There is extensive individual variation in the expression level of many genes in organisms from yeast to humans. In humans, the differences are smaller in monozygotic twins than among individuals of other relationships, suggesting a genetic contribution to the variation [1]. The data for Problem 1 came from studies of the genetic basis of variation in human gene expression [2,3].

## Methods

### Study subjects

The data provided to Genetic Analysis Workshop 15 (GAW15) were of several sorts. The basic collection was of data from large families, specifically 14 three-generation Centre d'Etude du Polymorphisme Humain (CEPH) Utah families (approximately 8 offspring per sibship and 14 individuals per family). The CEPH Utah families are the most uniform of the three-generation CEPH families (parents and grandparents are available) and cells are available for all four grandparents. The data provided were from 14 of these. In addition, gene expression data were provided from 30 "HapMap" trios: these are "grandparent-parent" trios that are partly included among those in the 14 families, plus approximately 12 additional grandparent-parent trios of CEPH Utah individuals. The 30 trios are also part of the International HapMap Project. The data included pedigree files with information on the structure of each family.

### Phenotypes

The expression level of genes expressed in lymphoblastoid cells (EBV-transformed B-cells) were obtained for the above subjects, using the Affymetrix Human Focus Arrays that contain probes for 8500 transcripts. For approximately 85 of the study subjects, array hybridizations were performed in duplicate. Data were provided for the 3554 genes for which we found greater variation among individuals than between replicates for the same individual. The cel files (raw image files) were provided, as well as slightly processed data (normalized data using the Affymetrix MAS software) for all array hybridizations.

### Genotypes

Genotypes of 2882 autosomal and X-linked SNPs for members of the 14 CEPH Utah families described above were provided. The genotypes were generated by The SNP Consortium [[4]; also ]. These marker genotypes were used for the mapping of gene expression phenotypes described in Morley et al. [2]. To make it possible to compare linkage results obtained in GAW15, the SNP genotype files that were used by Morley et al. [2] to generate the linkage results were also provided. These included the physical location of the SNP markers.

The genotypes for the HapMap subjects are freely available from the HapMap website . Because those data sets continue to be updated, they were not provided separately for GAW15.

## Discussion and possible analyses

The distinctive feature of these data is that genome scan data are provided for more than 3500 quantitative traits at one time. So instead of asking about properties of analysis methods for one phenotype at a time, once can ask about "operating characteristics" over a whole range of traits. Furthermore, because the phenotypes are in some sense all of one type (level of gene expression), it is natural to look for relationships among them, in a way that would not normally be appropriate for a collection of dissimilar traits, such as (for example) blood pressure, IQ, height, and serum glucose. The data offer an opportunity to look for genes whose expression appears to be co-regulated, and try to find where the determinants of these expression phenotypes map.

In addition, one might

1. Test methods for transforming expression data in a context where some answers are essentially "known."

2. Explore problems of multiple testing.

3. Explore replication by subsetting data, and/or by cross-validation and other post-hoc methods. Develop and use permutation tests appropriate for such a collection of data.

4. Look for interactions of chromosomal regions, or particular genes, etc.

## Conclusion

The data provided for Problem 1 offer a unique opportunity for analysis of genetics of variation in expression levels of a large number of genes. The data lend themselves to a wide variety of analyses, including mapping of determinants for individual expression phenotypes, testing for effects of multiple determinants, and technical aspects of microarray interpretation. The participants in GAW15 carried out a remarkable variety of approaches, with ingenious new ideas for learning from the data.

## Competing interests

The author(s) declare that they have no competing interests.
